# Transcranial Direct Current Stimulation on Parietal Operculum Contralateral to the Moving Limb Does Not Affect the Programming of Intra-Limb Anticipatory Postural Adjustments

**DOI:** 10.3389/fphys.2019.01159

**Published:** 2019-09-11

**Authors:** Silvia M. Marchese, Roberto Esposti, Francesco Bolzoni, Paolo Cavallari

**Affiliations:** Human Physiology Section of the Department of Pathophysiology and Transplantation, Università degli Studi di Milano, Milan, Italy

**Keywords:** tDCS, parietal operculum, intra-limb APAs, integration of voluntary movement and posture, human

## Abstract

Recent data suggest that the parietal operculum acts as an integration center within a multimodal network, originating from different primary sensory and motor cortices and projecting to frontal, parietal and temporal cortical hubs, which in turn govern cognitive and motor functions. Thus, parietal operculum might also play a crucial role in the integrated control of voluntary movement and posture. As a first step to test this hypothesis, the Anticipatory Postural Adjustments (APAs) stabilizing the arm when the index-finger is briskly flexed were recorded, on the preferred side, in three groups of 10 healthy subjects, before, during and after *CATHODAL* or *ANODAL* transcranial Direct Current Stimulation (tDCS, 20 min at 2 mA) applied over the contralateral Parietal Operculum (coPO). Results were compared to those obtained in a *SHAM* group. In agreement with literature, in the *SHAM* group the activation of the prime mover Flexor Digitorum Superficialis was preceded by an inhibitory APA in Biceps Brachii and Anterior Deltoid, and almost simultaneous to an excitatory APA in Triceps Brachii. The same pattern was observed in both the *CATHODAL* and *ANODAL* groups, with no significant tDCS effects on APAs amplitude and timing. Index-finger kinematics were also unchanged. These negative results suggest that the coPO does not disturb the key network governing APAs in index-finger flexion. Since it has been well documented that such APAs share many features with those observed in trunk and limb muscles when performing several other movements, we suggest that coPO may not be crucial to the general APA control.

## Introduction

Voluntary movements induce postural perturbations, which are usually counteracted by muscular activities involving muscles other than the prime mover. Some of them, the Anticipatory Postural Adjustments (APAs), develop well before the onset of the focal movement itself, and such anticipation witnesses that they are programmed in a feed-forward way (Belen’kii et al., 1967; Massion, 1998). APAs are tailored to the kinematical aspects of the primary movement and usually spread over different muscles, creating one or more fixation chains toward the available support points. Several studies described the APA chains that precede movements involving large masses, such as a shoulder flexion that produce a so large postural perturbation to threaten the whole-body equilibrium (Bouisset and Zattara, 1987). In these conditions, where it is important to avoid falling (Dakin and Bolton, 2018), APAs usually spread over different limbs so that they are referred to as *inter-limb* APAs. It has also been demonstrated that similar adjustments develop in the same limb when moving one of its distal segments, e.g., APAs in the arm when flexing/extending the hand (Aoki, 1991) and even when moving a very tiny mass as when flexing the index-finger at the metacarpophalangeal joint (Caronni and Cavallari, 2009). Such postural actions are referred to as *intra-limb* APAs. Considering that the perturbation produced when moving very tiny masses is irrelevant with respect to whole body equilibrium, it has been suggested that *intra-limb* APAs contribute to attain an higher precision of the focal movement (Bruttini et al., 2016).

The present study belongs to a broad line of research oriented to investigate how the APA control is organized. In fact, several studies showed the role of sensory and motor areas, including the primary and supplementary motor cortices, as well as subcortical structures like basal ganglia, cerebellum and spinal cord (Viallet et al., 1992; Palmer et al., 1994; Petersen et al., 2009; Bolzoni et al., 2012, 2015, 2018; Ng et al., 2013; Bruttini et al., 2015). Although it is still not well established whether the command for recruiting the prime mover muscles and that governing the postural muscles are separately processed or have a common origin, we provided evidence that a functionally unique motor command should drive both the prime mover and the muscles of the *intra-limb* APAs chain (Bruttini et al., 2014). Moreover, we have also found that the command splits before reaching the SMA (Bolzoni et al., 2015), since interfering with the excitability of this area affected the APAs but not the prime mover recruitment. In this context, it was of interest to move the investigation toward an higher-level integration center.

Considering that APAs are tuned depending on primary movement kinematics and that they adapt to the postural context, we chose a neural structure deeply involved in the integration of sensory-motor information. Recent studies highlighted the parietal operculum (PO) as a “hub,” in which converge several sensory-motor streams originating from different cerebral areas. The PO is “the cortical flap that covers the dorsocaudal part of the Sylvian fissure,” which may be divided into four cytoarchitectonical areas (OP1–OP4) (Eickhoff et al., 2006b; Cattaneo et al., 2015). Several studies focused on the role of PO in secondary sensory processes, highlighting its involvement in the integration of proprioceptive and tactile information within the framework of motor control (Milner et al., 2007; Sepulcre, 2014). Sepulcre et al. (2012) revealed how the connectivity of sensory and motor systems converge in a network that seems involved in linking external and internal information. PO is the crucial part, in this multimodal network, where visual, somatosensory and auditory functional streams converge; in turn, PO is connected to motor and premotor areas (Felleman and Van Essen, 1991). In this regard, it is also interesting to note that APAs are influenced by the availability of visual information (Esposti et al., 2017), which may indirectly point out a PO involvement. On these premises, we tested whether the PO contralateral to the moving limb contralateral Parietal Operculum (coPO) is involved in the control of *intra-limb* APAs associated to index-finger flexion movements.

In this aim, we modulated coPO excitability by using anodal and cathodal transcranial Direct Current Stimulation (tDCS), a technique which has been proved to selectively interfere with the excitability of many cortical structures involved in motor and cognitive processes (for a review see Brunoni et al., 2013), including PO (Fujimoto et al., 2017). Notably, the applied currents are usually sufficiently low to grant a focal stimulation but nevertheless they produced long-lasting effects in many cases (Brunoni et al., 2013).

Thus, by analyzing the effects of tDCS, it would be possible to test the hypothesis that coPO is involved in processing the *intra-limb* APAs associated to index-finger flexion, as well as whether in this area the motor commands to the prime mover and to postural muscles are split or still processed as a single functional stream.

## Materials and Methods

A total of 30 healthy volunteers (mean age ± SD: 27.5 ± 2.9 years, 20 males) were enrolled in this study. Oldfield questionnaire was used to ascertain handedness, resulting in only one left-handed participant. No subject had any history of neurological or orthopedic diseases, as well as of intake of drugs acting on the Central Nervous System. Participants provided their informed consent, but were kept completely unaware of the stimulation condition. The experimental protocol complied with the policies and principles contained in the Declaration of Helsinki and were approved by the Ethical Committee of the University of Milan (counsel 6/19).

**FIGURE 1:**
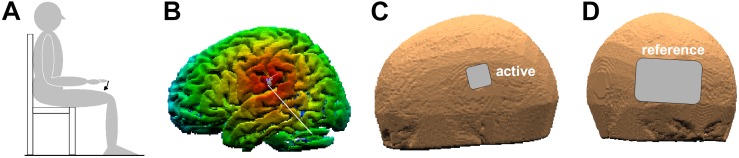
**(A)** Position of the experimental subject. The arrow indicates index-finger flexion with the preferred hand prone. **(B)** False-color map of the distance between the tip of the neuronavigator pointing stylus, positioned on the scalp, and the reconstructed brain surface. The white line points to the coPO, identified by means of its Talairach coordinates. **(C,D)** Position of the active and reference electrodes (3.16 × 3.16 cm and 8 × 12 cm, respectively).

Subjects were randomly assigned and equally distributed to one out of the three tDCS conditions (*ANODAL*, *CATHODAL*, and *SHAM*, see second-next paragraph). This between-subjects approach was chosen so as to exclude a carry-over effect due to multiple stimulations performed in the same subject on the same day. Subjects were tested on their preferred side. They were sitting on a chair with the non-preferred arm lying on an armrest, while the preferred upper arm was along the body, elbow flexed at 90° and the hand prone, lined-up with the forearm. The index-finger was kept extended and aligned with the hand, while all other fingers were hanging. During the experiment, subjects had to keep their back supported and both feet on the ground (Figure 1A). The experimenters adapted the set-up to each subject’s body size and supervised the subject’s position during the whole experimental session.

### Motor Task

At the beginning of the procedure, one of the experimenters held the preferred upper limb of the subject, who was instructed to exert a Maximal Voluntary Contraction (MVC) of each of the recorded muscles (see second-next paragraph), one at a time, for about 6–10 s, while the experimenter was monitoring the EMG. Then, after resting for about 10 min, the subject had to perform several sequences of 15 brisk flexion movements of the index-finger, at the metacarpophalangeal joint: two sequences, with about 30 s of rest in between, were performed just before applying tDCS (*Pre*), two at about half of the tDCS period (*Dur 10’*), two in the last minutes of full-current tDCS (*Dur 20’*) and two at 5, 10, and 20 min after tDCS end (*Post 5’*, *Post 10’*, *Post 20’*). Each movement was self-paced, after a beep (go signal, repeated every 7 s), so as to avoid any reaction time. In fact, the time between the go signal and the movement onset changed according to the subject’s will. No subject complained about fatigue.

### Neuronavigation and tDCS

Transcranial direct-current stimulation was applied by using a neuroConn^®^ DC-Stimulator Plus (model 0021) connected to two sponge electrodes, soaked with conductive gel. The active electrode (3.16 × 3.16 cm) was positioned on the scalp point closest to the PO of the non-preferred side (Figure 1C), as Mălîia et al. (2018) reported motor effects only when applying direct electrical stimulation on this side. The electrode positioning was guided by a neuronavigation system (SofTaxic Optic 2.0, see Figure 1B). In this aim, the coPO was identified by means of the average Talairach coordinates of its sub-areas PO1 and PO4, which are the closest to the subdural space (on the left: −52, −18.5, 22; on the right: 52, −18.5, 22.5; values obtained from MNI coordinates in Eickhoff et al. (2006a) and converted to Talairach according to Lacadie et al. (2008). The scalp position closest to coPO was then identified using the neuronavigation pointing stylus (Figure 1B). A much larger reference electrode (8 × 12 cm, so as to be functionally inefficient) was instead placed on the forehead over the contralateral supraorbital area (Figure 1D). Both electrodes were fixed by elastic bands.

Ten subjects underwent *ANODAL* tDCS, ten underwent *CATHODAL* and ten *SHAM*. Anodal and cathodal tDCS started with a 60 s fade-in period, followed by 20 min DC at 2 mA and a 30 s fade-out. In sham configuration, instead, the 60 s fade-in was immediately followed by the 30 s fade-out. The resulting current density (2 A/m^2^) was much lower than the safety limit (25.46 A/m^2^) reported on humans by Bikson et al. (2009) and even smaller than the minimal current density (142.9 A/m^2^, Liebetanz et al., 2009) that might induce brain lesion in the rat. We actually applied a similar setup in one of our previous studies (Bolzoni et al., 2017) and none of the subjects reported unpleasant sensations or could recognize the DC polarity. Throughout the experiment, it was checked that scalp impedance was constant and never exceeded 5 kΩ (range 1.2–4.2 kΩ).

### Movement and EMG Recordings

Flexion-extension of the index-finger at the metacarpophalangeal joint was recorded on the preferred side by a strain-gauge goniometer (mod. F35, Biometrics Ltd^®^, Newport, United Kingdom) stuck on the skin with hypoallergenic tape. Angular signal was DC amplified (P122, Grass Technologies^®^, West Warwick, RI, United States) and gain was calibrated before each experiment.

EMG signals were recorded by pairs of pre-gelled surface electrodes (H124SG, Kendall ARBO, Tyco Healthcare, Neustadt/Donau, Germany) placed on the prime mover Flexor Digitorum Superficialis (FDS) of the preferred upper limb and from some of the ipsilateral muscles involved in stabilizing the arm (Caronni and Cavallari, 2009): Biceps Brachii, Triceps Brachii, and Anterior Deltoid (BB, TB, and AD). The inter-electrode distance was 24 mm and electrode placement for BB, TB, and AD followed the SENIAM guidelines (Hermens and Freriks, 1999). The same general approach was adopted for FDS, for which muscle no specific SENIAM guidelines are available: the subject kept his preferred arm and forearm in the experimental position while repeatedly flexing one finger at a time. Meanwhile, the experimenter palpated the forearm, so as to feel the contraction of the FDS belly, on which the electrodes were placed. Recordings selectivity was verified by checking that activity from the recorded muscle, during its phasic contraction, was not contaminated by other muscular sources. EMG signals were amplified (IP511, Grass Technologies^®^, West Warwick, RI, United States) with a 1–20 k gain and a band-pass filter at 30–1000 Hz, so as to minimize movement artifacts and high frequency noise.

Conditioned goniometric and EMG analog signals were then sampled at 1 kHz, with an anti-aliasing low-pass filter at 500 Hz and a 12-bit resolution (A/D board model PCI-6024E, National Instruments^®^, Austin, TX, United States).

### Data Analysis

All the EMG traces were digitally rectified, then the traces collected while moving the index-finger were expressed in % of the highest average EMG value recorded for 1 s during the subject’s MVC monitoring.

For each EMG and goniometric variable, the 30 traces recorded in the two sequences *Pre* tDCS were time-aligned to the point (*trigger*) in which finger flexion reached 15° with respect to its resting position (mean value from 1 to 0.1 s before the go signal), and averaged. Such trigger choice actually granted the time-alignment precision, as it was verified that at 15° flexion the index-finger was moving at more than 50% of its peak velocity. The resulting averaged trace extended from 2 s before to 0.3 s after the trigger. The same procedure was applied for the 30 traces obtained in *Dur 10’*, *Dur 20’*, *Post 5’*, *Post 10’*, and *Post 20’*. All subsequent measurements were taken on the averaged traces, and visually validated.

The first measurement regarded the onset of index-finger movement. The mean level of the signal recorded from 1 to 0.5 s prior to the *trigger* (reference period) was subtracted from the averaged trace, then a software algorithm searched the first time point in which the trace fell below −2 SD of the signal in the reference period and remained below that level for at least 50 ms. When the criterion was met, the algorithm searched backward the point in which the trace started to deviate from the mean reference value. Movement amplitude and duration were measured, respectively, as the amplitude and timing difference between the peak flexion of index-finger and the onset of its movement.

For each average EMG trace, the period from 1 to 0.5 s before movement onset (where no voluntary activity in FDS nor EMG changes in postural muscles occurred) was assumed as reference. The trace was integrated (time constant = 11 ms) and the mean reference level was subtracted from it; then the onset of an excitatory or inhibitory EMG change was identified by the above-described software algorithm, setting the threshold at +2 SD or −2 SD of the reference signal, respectively. The search was stopped at the onset of index-finger movement, so as to avoid any effect due to re-afferentation triggered by the focal movement. All timings were expressed as latencies with respect to FDS onset, with negative values representing time-advances. Finally, the amplitude of the EMG changes were measured as the mean level in the time-window from the onset of the EMG change to the onset of index-finger movement.

For each measured variable, a two-way ANOVA was applied to test for the effects of tDCS *polarity* (*SHAM* vs. *ANODAL* vs. *CATHODAL*) and *time* (*Pre* vs. *Dur 10’* vs. *Dur 20’* vs. *Post 5’* vs. *Post 10’* vs. *Post 20’*; repeated measurements factor), as well as their *interaction*. For all tests, statistical significance was set at *p* < 0.05 and the effect size was calculated by the partial eta square (η^2^_p_).

With this statistical design, the meaningful effect to be searched for is whether the within-subjects changes in *time* (*Pre* vs. *Post 20’*) are different among the three *polarities*, which is the *interaction* effect. Power analysis showed that the present design has 87% power to detect an interaction effect as low as η^2^_p_ = 0.13. Such value is half the effect size of the minimum significant difference that we found in a similar experiment in which tDCS modulated SMA excitability (Bolzoni et al., 2015).

## Results

**FIGURE 2:**
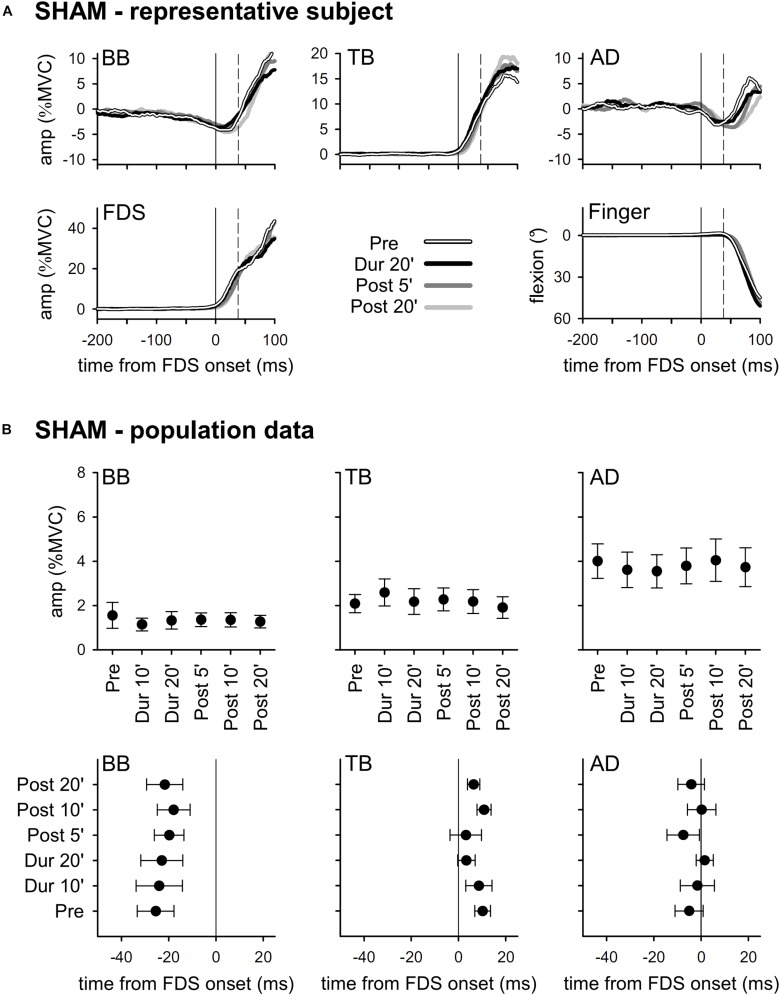
In the **(A)**, rectified EMG and kinematics traces from a representative subject, who underwent *SHAM* tDCS (shades of black). Averages of 30 movement trials, recorded immediately before tDCS (*Pre*), in the last minutes of the “virtual” full-current period (*Dur 20’*), and at 5 and 20 min after it (*Post 5’* and *Post 20’*). At all the time-points, the onset of activity (solid vertical line) in the prime mover Flexor Digitorum Superficialis (FDS) was accompanied by inhibitory APAs in Biceps Brachii (BB) and Anterior Deltoid (AD), and by an excitatory APA in Triceps Brachii (TB), which always preceded movement onset (dashed vertical line). Note how at each time point the traces are at all comparable, indicating that repeating the motor task had no effect on APAs, prime mover recruitment and focal movement kinematics. In the **(B)**, mean (±SE) amplitude and latencies of APAs recorded in the BB, TB and AD muscles of all subjects of the *SHAM* group. No significant changes occurred among the different time-points (*Pre* vs. *Dur 10’* vs. *Dur 20’* vs. *Post 5’* vs. *Post 10’* vs. *Post 20’*), confirming the stability of APAs.

The upper part of Figure 2 illustrates the mean integrated EMG and kinematics traces obtained from a representative subject who underwent *SHAM* tDCS. Traces are averages of 30 movement trials, recorded immediately before tDCS application (*Pre*), in the last minutes of “virtual” full-current tDCS (*Dur 20’*), and after 5 and 20 min of “virtual” recovery (*Post 5’* and *Post 20’*). In full agreement with the literature (Caronni and Cavallari, 2009), in *Pre* the FDS onset (solid vertical line) was accompanied by an inhibition in BB and AD, and by an excitation in TB. Such EMG changes always occurred before movement onset (dashed vertical line) and acted so as to stabilize the arm against the perturbation due to finger flexion, thus being classified as APAs. It is also apparent that the traces recorded in *Dur 20’*, *Post 5’*, and *Post 20’* were at all comparable to those recorded in *Pre.* The lower part of Figure 2 reports the mean values of APAs amplitude and latency obtained in the whole population. Note that the APAs recorded during and after *SHAM* tDCS were at all comparable to those recorded in *Pre*; this confirms that repeating the motor task had no effect, actually excluding any contribution of fatigue.

**FIGURE 3:**
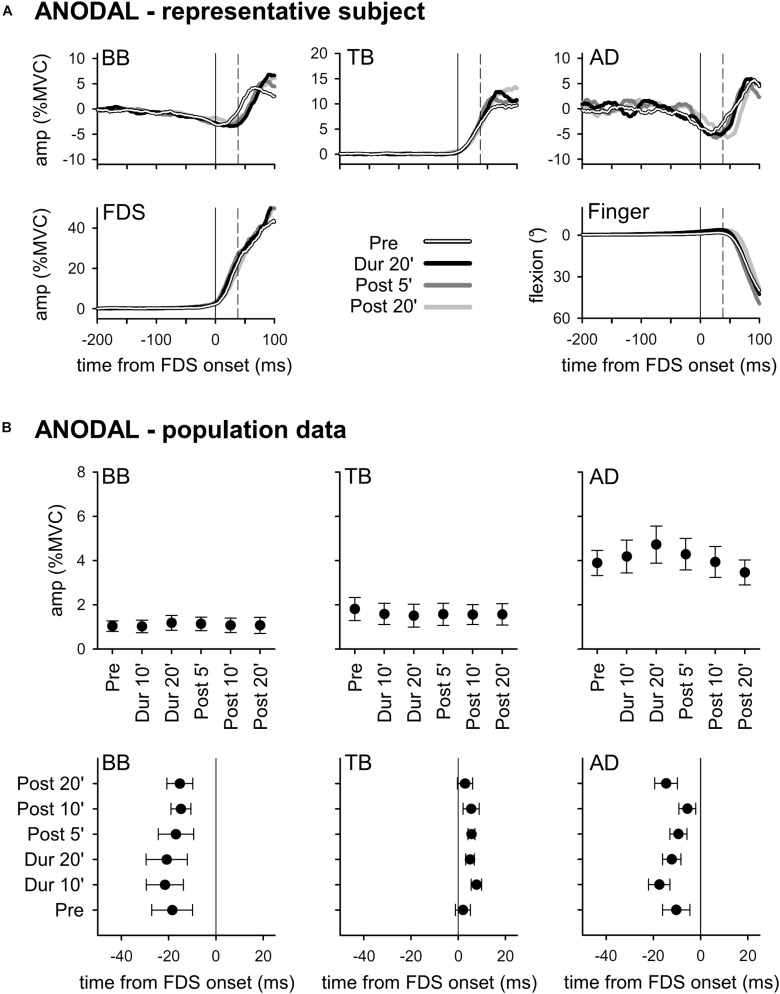
Traces from a representative subject **(A)**, as well as mean (±SE) amplitude and latencies of APAs recorded in the BB, TB, and AD muscles of all subjects **(B)** who underwent *ANODAL* tDCS. Same layout as in Figure 2. It is apparent that tDCS had no effect on APAs, as no significant changes occurred among the different time-points (*Pre* vs. *Dur 10’* vs. …vs. *Post 20’*).

**FIGURE 4:**
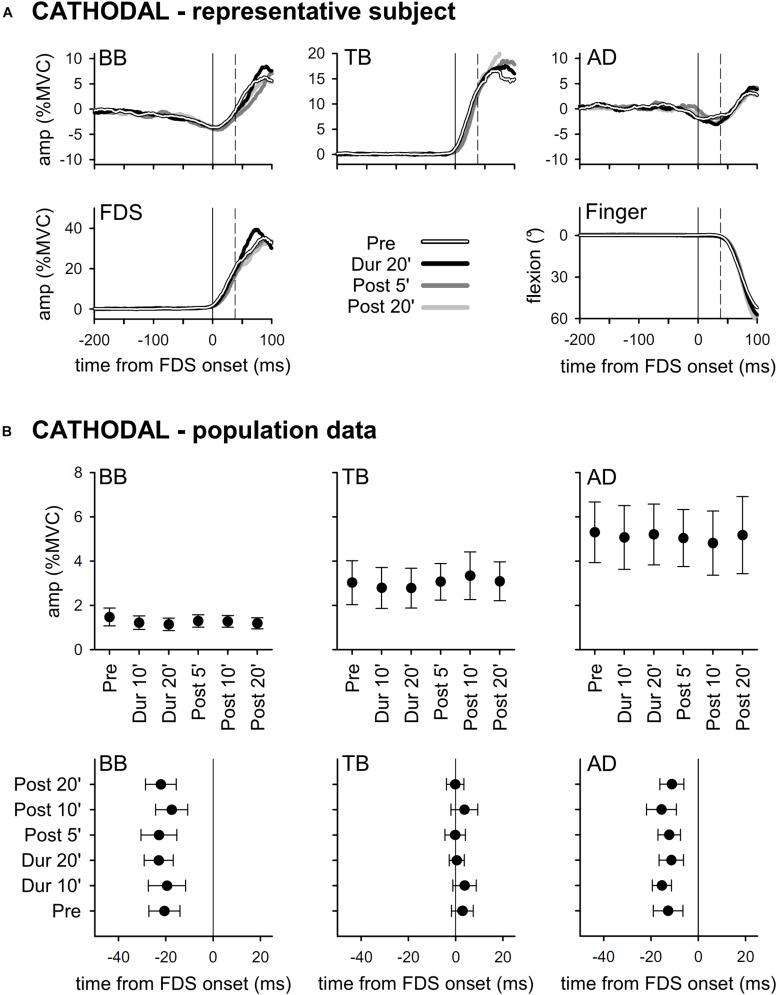
Traces from a representative subject **(A)**, as well as mean (±SE) amplitude and latencies of APAs recorded in the BB, TB, and AD muscles of all subjects **(B)** who underwent *CATHODAL* tDCS. Same layout as in Figure 2. Also with this polarity, tDCS had no effect on APAs (no significant changes *Pre* vs. *Dur 10’* vs. …vs. *Post 20’*).

Results obtained with *ANODAL* and *CATHODAL* tDCS were at all comparable to those recorded in *SHAM* condition (Figures 3, 4), indicating that applying current of either polarity had no effect on APAs amplitude or latency. Such finding was also supported by statistics: two-way ANOVAs failed to highlight any significant *interaction* (in all muscles, *F*_10__,__135_ ≤ 1.604, *p* ≥ 0.11, η^2^_p_ ≤ 0.106). The same was true for the main effects of *time* (*F*_5__,__135_ ≤ 1.326, *p* ≥ 0.26, η^2^_p_ ≤ 0.047) and *polarity* (*F*_2__,__27_ ≤ 1.641, *p* ≥ 0.21, η^2^_p_ ≤ 0.108). Finally, statistics witnessed that tDCS had no effect on amplitude of FDS recruitment and index-finger kinematics too, as the two-way ANOVAs failed to find any significant effect (*interaction F*_10__,__135_ ≤ 1.392, *p* ≥ 0.19, η^2^_p_ ≤ 0.093; *time F*_5__,__135_ ≤ 1.577, *p* ≥ 0.17, η^2^_p_ ≤ 0.055; *polarity F*_2__,__27_ ≤ 1.159, *p* ≥ 0.33, η^2^_p_ ≤ 0.079).

## Discussion

According to our results, tDCS of either polarity applied over the coPO does not affect amplitude or latency of *intra-limb* APAs associated to index-finger flexion. Before concluding that coPO is not involved in the control of such APAs, some considerations are worthy. First, it could be argued that tDCS duration and current intensity or density were insufficient for modulating PO excitability. This could be reasonably excluded, as Fujimoto et al. (2017) obtained significant differences in tactile orientation discrimination when applying tDCS over PO, with the same current intensity but a 2.5 times smaller current density and for a duration 5-minute shorter than in our work. Moreover, if one takes into account the electric field simulations published by Fujimoto in the same paper, it is apparent that 2 mA tDCS is more than sufficient to alter the electric potential over the area of interest. Second, problems in locating our active electrode over the target area should be excluded. Indeed, despite it is impossible to check having found the right scalp position by, e.g., eliciting overt motor responses using transcranial magnetic stimulation, we feel confident that our neuronavigation system granted a reasonably good positioning. Third, it could be objected that more subjects are needed to highlight tDCS effects. However, in a similar study (Bolzoni et al., 2015), we applied tDCS over SMA and gathered evident results with a comparable number of subjects, while changes observed in the present study were inconsistent (Figures 3, 4). Finally, from a purely speculative perspective, it may be hypothesized that a significant difference would have been observed if stimulating the PO ipsilateral to the moving finger, or both POs. This hypothesis would contrast with the general scheme of motor pattern generation, which classically involves the cerebral hemisphere contralateral to the moving limb and the cerebellar hemisphere ipsilateral to it. In this regard, it should be also recalled that direct electrical stimulation of PO evoked motor effects only when applied to the left side, and that motor and sensory effects were mainly (90%) on the right side (Mălîia et al., 2018). In any case, it cannot be *a priori* excluded that the earlier phases of the motor act processing might involve both hemispheres. Clearly, a definite answer could be obtained only by direct testing. Should also these last possibilities fail, our search for an area in which the motor command to prime mover and postural muscles are still functionally unique (as defined in the Introduction) will have to address other structures.

On the other side, the conclusion that coPO stimulation does not disturb the control of APA associated to index-finger flexion does not contrast with the principal role that literature assigns to such structure. Indeed, the PO seemingly exerts its influence in the earlier strategic phase of selecting the motor goal, rather than in the planning of the motor act, where the motor program for the prime mover and the related APA chains are defined (Tunik et al., 2008; Woods et al., 2014; Valyear and Frey, 2016). For this reason, PO may not directly affect the integration of voluntary movement and posture, thus leaving these APAs unchanged. The absence of any significant effect on prime mover recruitment and index-finger kinematics is at all consistent with the fact that in our experiments the motor goal is intrinsically defined by the experimental task and thus its selection had already occurred well-before tDCS application.

Several studies suggested that PO has an important role in working memory and tactile learning (Jäncke et al., 2001), indeed, this neural structure seems to contain haptic memory information and it might be more important for object-directed motor behavior (Maule et al., 2015) rather than in planning the motor act, as it has been demonstrated for its neighbor frontal operculum (Tunik et al., 2008). Moreover, the PO network seems to modulate auditory-sensorimotor control, by mediating multimodal integration (Tanaka and Kirino, 2018), as well as orofacial muscles movements (Grabski et al., 2012), probably for phonation purposes. So, it may be argued that the contribution of PO concerns more specific motor actions and learning-memory rather than the motor planning. Our observation that no alteration occur in APAs associated to index-finger flexion when modulating coPO excitability is consistent with the above reasoning.

Lastly, since it is well documented that the index-finger APAs and those preceding other limb movements (*intra-* and *inter-limb* APAs, for a review see Cavallari et al., 2016) share not only their principal behavioral features but also their neural control structures, it may be advisedly suggested that coPO may not be crucial to the APA control in general.

## Conclusion

The well-known role of PO in sensory-motor integration processing led us to inquire its possible involvement in postural control during index-finger flexion, a task which notably adapts to the perceived postural context, as it has been shown to occur for more general APAs. However, the present results seem to exclude such an hypothesis. Indirectly, this supports literature data that place PO within the sensorimotor integration network for selecting the motor goal. In order to definitely exclude the role of PO in APA control, future experiments should apply tDCS over the PO ipsilateral to the moving upper limb and/or bilaterally over the two POs. If also those trials will fail, the search for an area in which the motor command to prime mover and postural muscles are still functionally unique will have to move to other structures.

## Data Availability

The datasets generated for this study are available on request to the corresponding author.

## Ethics Statement

The studies involving human participants were reviewed and approved by Ethical Committee of the University of Milan (counsel 6/19). The patients/participants provided their written informed consent to participate in this study.

## Author Contributions

PC conceived the study. SM, RE, and FB conducted the experiments and analyzed the results. All authors contributed to drafting the manuscript and approved the final version.

## Conflict of Interest Statement

The authors declare that the research was conducted in the absence of any commercial or financial relationships that could be construed as a potential conflict of interest.
